# Private Arrangements for Medical Benefit

**Published:** 1913-02-08

**Authors:** 


### PRIVATE ARRANGEMENTS FOR MEDICAL BENEFIT.

The publicity which has lately been afforded in Parliament and the press to the attempts made by various National Insurance Committees to prevent all insured persons from taking advantage of those provisions in the Act which allow them to "make their own arrangements for medical benefit" can do nothing but good. The paragraph on "Form Med. 21," which we published last week, the disclosures of Mr. E. B. Turner on p. 514 of this issue, and the debate at the meeting of the Middlesex Insurance Committee reported in the public press of February 5, all show that the game is not being fairly played. A deliberate and apparently concerted attempt is being made to render this section of the Act a dead-letter; and it is evident that this is being done as a way of punishing those doctors who have not yet bowed the knee and broken the pledge, and as a means of rewarding those who have been cajoled or browbeaten into submission.

In every official statement about applications for permission to contract out of the panel system of medical benefit, the offensive phrase is used that so many applications have been received "purporting to come from insured persons." Since such requests are necessarily signed by those who make them, this phrase becomes meaningless except as an indication of the hostility of the Committees to "free choice of doctor" in any real and effective sense. The discussion of the Middlesex Committee is of especial interest in this connection. The chairman is Mr. Glyn-Jones, a compatriot and ardent supporter of Mr. Lloyd George; and he made himself conspicuous a month or two ago by ingenious sophistries designed to cause the local doctors to break their pledges. Successful to a considerable degree in these efforts, he is now confronted with numerous applications from insured persons to make their own arrangements. These he characterises as attempts "to evade the scheme set up by the Committee" of which he is chairman, and he does not propose to allow them. Supported by the representatives of the approved societies (who constitute the majority of each Committee), Mr. Glyn-Jones is able to ignore both insured persons and ordinary fair play; and that is what he is proceeding to do. Only in exceptional cases he has decided will such permission be granted; and not at all when the insured want to be attended by those who, after joining, have withdrawn from the panels.

This high-handed conduct is a specimen of what is going on in other districts; and it is quoted, not because it is exceptional, but simply because there is no attempt at concealment, which shows that in Middlesex the medical profession is held in contempt by the Committee, and the insured are regarded as a negligible quantity. But we are quite certain that the people are quietly taking note of the way in which they are being used as pawns in the party game, and Mr. T. C. Winn, of Hackney, who is a panel doctor, has extracted from the London Insurance Committee what is virtually a statement that he may neglect his patients when business is brisk, but that at all costs he must keep his books up to date. This astonishing proposition is one which the insured may be trusted to take note of.

